# Hyperviscosity Syndrome in Paraprotein Secreting Conditions Including Waldenstrom Macroglobulinemia

**DOI:** 10.3389/fonc.2020.00815

**Published:** 2020-05-19

**Authors:** Allison Weaver, Samuel Rubinstein, Robert F. Cornell

**Affiliations:** Division of Hematology and Oncology, Department of Internal Medicine, Vanderbilt University Medical Center, Nashville, TN, United States

**Keywords:** hyperviscosity, plasma exchange, Waldenstrom, lymphoma, chemotherapy

## Abstract

Hyperviscosity syndrome is a serious complication associated with high levels of paraproteins in patients with hematological malignancies. Therapeutic advances in disease control may reduce the incidence of hyperviscosity syndrome; however, management of acute cases requires an understanding of key symptoms and prompt treatment to mitigate serious consequences.

## Introduction

Hyperviscosity syndrome (HVS) occurs in patients with hematological malignancies that secrete high levels of paraprotein (i.e, IgM, IgG, and IgA). It is most commonly observed in Waldenstrom macroglobulinemia (WM), affecting 10–30% of patients, and has been reported in 2–6% of patients with multiple myeloma (1–6). HVS has also been recently reported in a patient with splenic marginal zone B cell lymphoma (IgM-κ) (7). HVS is a potentially life-threatening medical emergency that requires prompt recognition and intervention.

## Paraprotein Levels and Hyperviscosity Syndrome

Viscosity can be measured in absolute terms in centipoise (cP), or in relative terms compared to the viscosity of water (0.894 cP). A typical serum viscosity for a healthy patient is 1.5 cP, or 1.7 relative to water. The increased viscosity of serum relative to water relates primarily to its protein content. In contrast to most serum proteins, which are spherical in shape, immunoglobulins are relatively large but also linear in shape; thus, when they travel through the serum, they spin around their longitudinal axis, increasing serum viscous drag, and therefore viscosity. Serum viscosity is correlated with immunoglobulin levels for patients both with and without monoclonal gammopathies (8), but the concentration of immunoglobulins required to significantly increase viscosity depends on the specific type of paraprotein. IgM is pentameric and very large in size (970 kDA), and serum viscosity can increase significantly with IgM levels as low as 3 g/dL, and IgM levels of 6 g/dL or higher are associated with rapid development of hyperviscosity, with a median time to symptomatic HVS of 3 months (9, 10). IgA is smaller (320 kDA) than IgM but circulates as a dimer and is associated with increased viscosity at levels of 6 g/dL or greater (5, 11). IgG is relatively small (180 kDA) and can require levels as high as 10 g/dL to produce significant changes in viscosity, except for the IgG3 subtype, which has a tendency to aggregate and can increase viscosity at lower levels, perhaps due to its elongated hinge region and resultant increases in Fc-Fc interaction (9, 12, 13). Even in the context of a paraprotein-excreting hematologic malignancy, the risk of HVS is relatively low until the serum viscosity increases above 4 cP (9). It should be noted that, although the likelihood of HVS increases at higher levels of immunoglobulin, there is no discrete cutoff in any circumstance, and HVS should be considered for patients with characteristic symptoms and evidence of a paraprotein-secreting hematologic malignancy.

## Symptoms and Management of Hyperviscosity Syndrome

The predominant symptoms of HVS are mucocutaneous bleeding, ophthalmologic, and neurologic (Table 1). Hemorrhage typically occurs in small venules associated with increased viscosity in areas with minimal supporting tissue, such as the nose and oral cavity. Epistaxis is a common presenting symptom, and if present in a patient with a possible paraproteinemia, should prompt additional evaluation for HVS, particularly fundoscopic examination, as evidence of ocular HVS can be present without visual symptoms and is a treatment indication. Ophthalmologic symptoms include blurred or double vision, retinal hemorrhage. Due to the high prevalence of ophthalmologic manifestations of HVS, a fundoscopic examination should be performed in any patient with suspected HVS or with a serum IgM > 3 g/dL. Characteristic retinal vein dilation with tortuous “sausage link” appearance on retinal veins can be seen. Other ophthalmic findings may include flame hemorrhages, papilledema, exudates, and microaneurysms. The most severe ophthalmologic manifestation of HVS is central retinal vein occlusion, which can result in irreversible vision loss and has been reported for patients with IgM and non-IgM paraproteinemias (14). Neurologic manifestations can range from relatively mild headache and lightheadedness to seizures and coma (4, 9, 15–20).

**Table 1:** Clinical manifestations of hyperviscosity syndrome.

Central Nervous System	Headache Dizziness and vertigo Impaired consciousness Somnolence Tinnitus and impaired hearing Ataxia Seizure
Ophthalmologic	Blurred or loss of vision Diplopia Retinal vein occlusion Papilledema Retinal hemorrhage
Mucocutaneous	Epistaxis Gingival bleeding Mucocutaneous bleeding Gastrointestinal bleeding
Cardiovascular	High-output cardiac failure Renal impairment Priapism
Other	Fatigue Malaise Shortness of breath

Plasmapheresis has been used effectively for management of HVS associated with WM since the 1950's. Despite the lack of randomized trials, this procedure rapidly reverses the clinical symptoms of hyperviscosity. Therapeutic plasma exchange (TPE) removes large-molecular-weight substances from patient plasma, including paraproteins, with the return of all cellular components to the patient. This is done by passing venous blood through an extracorporeal blood centrifugal separation device, which allows for shunting of plasma for removal. The remaining blood components are returned to the patient along with a short-acting anticoagulant, such as citrate. TPE is usually carried out using an automated blood cell separator to ensure fluid balance and maintain a normal plasma volume. Central line placement is used to allow adequate blood flow. Typically, 30–40 mL/kg of plasma (1–1.5 plasma volumes) are removed at each procedure and replaced with isotonic 4.5 or 5.0% human albumin solution. Some services substitute 25–50% of replacement volume with 0.9% saline (21). A single plasma exchange reduces viscosity 20–30% (22). Approximately 75% of IgM is intravascular and therefore only one or two sessions of TPE are necessary to reduce HVS in WM (23). However, in cases of IgG-associated disease, 45–65% of IgG is intravascular. The turnover rate from extravascular to intravascular IgG is only 1–3% per hour, thus, consecutive TPE every 24–48 h for 4–5 days may be necessary to reduce hyperviscosity in IgG-associated disease (24). In the absence of concurrent chemotherapy, IgG-associated disease can experience a rebound phenomenon reaching or exceeding pre-TPE levels due to persistent paraprotein production (23). Caution should be taken to avoid excessive packed red blood cell or platelet transfusions until TPE has successfully reduced hyperviscosity (15).

In general, TPE is a safe procedure, with a severe adverse event rate of 1.0% in a European registry study of over 7,000 patients undergoing filtration-based exchange (25, 26). Potential complications of TPE include hypotension, allergic reaction to replacement fluid, hypofibrinogenemia, and metabolic abnormalities caused by citrate use. While repeated plasmapheresis regimens can alleviate HVS symptoms, chemotherapy should be used for long-term disease control and consequent reduction of serum paraproteins.

## Chemotherapy Management for Hyperviscosity Syndrome

**Figure 1:**
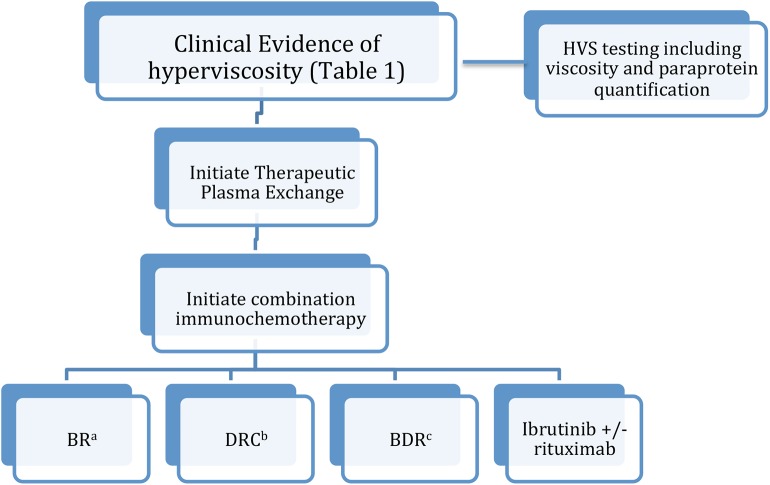
Treatment algorithm for hyperviscosity syndrome in WM. (a) Bendamustine/rituximab. (b) Dexamethasone/cyclophosphamide/rituximab. (c) Bortezomib/dexamethasone/rituximab.

As discussed previously, while hyperviscosity syndrome remains overall a rare occurrence, it can occur in up to a third of WM patients in their lifetime (1–6). As such, prompt treatment of hyperviscosity and the underlying WM is paramount in preventing the morbidity and mortality associated with this condition. Plasma exchange has been demonstrated to be effective in rapid symptomatic improvement as well as reversal of retinopathy (20). Plasma exchange can be continued until symptomatic improvement in HVS with concurrent initiation of chemotherapy (27). Chemotherapy selection is key in maintaining response and preventing HVS recurrence. Most data supports the use of a multidrug regimen in the setting of HVS (Figure 1).

Significant progress has been made in the treatment WM and lymphoplasmacytic lymphoma (LPL), including the transition to routine use of anti-CD20 monoclonal antibodies (e.g. rituximab or ofatumumab), alkylating agents (e.g., cyclophosmide or bendamustine), nucleoside analogs (e.g., cladribine or fludarabine), proteasome inhibitors (e.g., bortezomib and carfilzomib), and bruton tyrosine kinase inhibition (i.e., ibrutinib) (28).

Current chemotherapy strategies generally center on rituximab therapy in combination with other systemic agents. Overall, there is a dearth of clinical trial data comparing the different regimens, and regimen choice often depends on clinician and patient preference as well as side effect profiles (29). Combination bendamustine/rituximab (BR) is appropriate for first-line therapy, with a phase 3 clinical trial demonstrating prolonged PFS and better tolerance compared to R-CHOP for patients with indolent malignancies including LPL (30). However, given the risk of rituximab induced IgM flare, the addition of rituximab is recommended only when the serum IgM level is <4,000 mg/dL. Bortezomib/rituximab/dexamethasone (BDR) and cyclophosphamide/rituximab/dexamethasone (DRC) are other commonly-used first line regimens. A meta-analysis of 22 WM trials found that these combinations result in comparable response rates and side effect profiles (31). Ibrutinib/rituximab combination is also an option given the relative tolerability of this regimen and ibrutinib's ability to rapidly reduce IgM levels in a matter of weeks (27, 32). There is also limited frontline data on the use of ibrutinib monotherapy, although more investigation is needed prior to recommending it for patients with a history of HVS due to WM/LPL (33).

Autologous and allogeneic hematopoietic cell transplants (alloHCT) are less commonly used to treat patients with WM/LPL but may be a viable option for disease management in select cases as a long term means of disease control and prevention of HVS. An analysis of 144 patients with WM revealed that 46% of patients achieved progression-free survival 5 years after alloHCT, with a rate of relapse of 24% (34).

While advances in disease management are expected to reduce the incidence of HVS in this patient population, HVS remains a significant complication that must be carefully monitored. The treating clinician has a variety of frontline regimens to choose from, with consideration of side effect profiles and patient performance ultimately guiding treatment in the absence of strong clinical evidence supporting a specific regimen.

## Paradoxical IgM Increase Following Rituximab Treatment

Initial rituximab therapy is associated with an increase in serum IgM concentrations in 30–70% of patients. Peak IgM was observed at a mean of 4 weeks, ranging from 1 to 8 weeks, from the start of rituximab use (35, 36). TPE should be performed before rituximab-based therapy in patients with an IgM level of ≥ 4,000 mg/dL or serum viscosity > 3.5 cp (20, 37). Thereafter, IgM should be closely monitored after rituximab initiation and repeated TPE should be considered in patients with recurrent symptomatic hyperviscosity. As rituximab-based treatment regimens become more broadly used for WM, patients should be followed closely for evidence of increased IgM production and dangerous changes in serum viscosity.

## Summary and Conclusions

Hyperviscosity syndrome due to high levels of paraprotein is a serious, and potentially life-threatening, clinical complication most commonly observed in patients with WM. Plasmapheresis remains an important tool for reducing the symptoms of HVS in emergent cases and reducing serum viscosity prior to rituximab initiation. Advances in chemotherapy regimens for WM will reduce the incidence of acute HVS through long-term management of underlying disease.

## Author Contributions

RC responsible for manuscript design. The final article was critically reviewed and approved for publication by RC, SR, and AW. All authors contributed to the writing and editing process and collaborated on the manuscript.

## Conflict of Interest

The authors declare that the research was conducted in the absence of any commercial or financial relationships that could be construed as a potential conflict of interest.
